# Limitations to intergenerational inheritance: subchronic paternal stress preconception does not influence offspring anxiety

**DOI:** 10.1038/s41598-020-72560-z

**Published:** 2020-09-29

**Authors:** K. A. Fennell, R. G. G. Busby, S. Li, C. Bodden, S. J. Stanger, B. Nixon, A. K. Short, A. J. Hannan, T. Y. Pang

**Affiliations:** 1 The Florey Institute of Neuroscience and Mental Health, 30 Royal Parade, Parkville, VIC 3052 Australia; 2 Institute of Applied BioSciences and Chemistry, HAN University of Applied Sciences, Nijmegen, The Netherlands; 3 Discipline of Biological Sciences, Priority Research Centre for Reproductive Science, School of Environmental and Life Sciences, University of Newcastle, Callaghan, NSW 2308 Australia; 4 Pregnancy and Reproduction Program, Hunter Medical Research Institute, New Lambton Heights, NSW 2305 Australia; 5 Department of Pediatrics, University of CA – Irvine, Irvine, CA USA; 6 Department of Anatomy and Neuroscience, The University of Melbourne, Parkville, VIC 3010 Australia

**Keywords:** Endocrine system and metabolic diseases, Stress and resilience

## Abstract

Independent studies have observed that a paternal history of stress or trauma is associated with his children having a greater likelihood of developing psychopathologies such as anxiety disorders. This father-to-child effect is reproduced in several mouse models of stress, which have been crucial in developing a greater understanding of intergenerational epigenetic inheritance. We previously reported that treatment of C57Bl/6J male breeders with low-dose corticosterone (CORT) for 28 days prior to mating yielded increased anxiety-related behaviours in their male F1 offspring. The present study aimed to determine whether subchronic 7-day CORT treatment of male mice just prior to mating would be sufficient to induce intergenerational modifications of anxiety-related behaviours in offspring. We report that subchronic CORT treatment of male breeders reduced their week-on-week body weight gain and altered *NR3C1* and *CRH* gene expression in the hypothalamus. There were no effects on sperm count and glucocorticoid receptor protein levels within the epididymal tissue of male breeders. Regarding the F1 offspring, screening for anxiety-related behaviours using the elevated-plus maze, light–dark box, and novelty-suppressed feeding test revealed no differences between the offspring of CORT-treated breeders compared to controls. Thus, it is crucial that future studies take into consideration the duration of exposure when assessing the intergenerational impacts of paternal health.

## Introduction

In the past decade, there has been a growing body of evidence that a parental life history of exposures to severe stress and trauma is a significant risk factor for psychopathologies developing in the next generation^1^. One of the best-regarded examples of this is the series of studies of the adult children of Holocaust survivors. Contingent on whether their parent suffered from Holocaust-related PTSD, these individuals were more likely to self-report post-traumatic stress disorder (PTSD)-like symptoms and have features of a dysregulated hypothalamus–pituitary–adrenal (HPA) axis^2–4^. Separately, the severity of PTSD for first responders to the World Trade Center attacks is also reported as a significant predictor for their young children to develop behavioural problems^5,6^. The children of pregnant women who were indirectly affected by the events of 9/11 are also reported to display anxiety-associated behavioural impairments, such as a poorer negative response to novelty at 9 months of age^7^. While these reports and others collectively suggest that a parental history of trauma or severe stress exposure has adverse impacts on their children (even from a very early age), the phenomenon of parent-to-child intergenerational inheritance in humans remains controversial. One major reason for this is that if intergenerational shifts were constant, then large variations in human health would be observed across time (especially in post-war generations) and there is no evidence of this occurring.

Preclinical studies utilising different mouse models of stress have provided consistent evidence supporting the clinical observations that paternal trauma or severe stress preconception can induce phenotypic modifications in offspring. For example, in the chronic social defeat stress model of PTSD and the chronic variable stress model, adult offspring sired by stressed males displayed pro-anxiety and depressive-like behaviours despite the offspring themselves never having been exposed to external sources of stress^8–10^. The behavioural deficits were accompanied by dysregulation of the HPA axis with suppression of their corticosterone (CORT) response to stress. The HPA axis is a highly conserved regulatory system^11,12^ and its dysregulation is implicated in the pathogenesis of various conditions including metabolic syndromes^13^ and neurodegenerative diseases^14^. Our group previously modelled generalised daily stress and chronic elevation of HPA axis activity by supplementing male C57Bl/6J breeders with low-dose CORT for 28 days prior to mating. In the process, we discovered transgenerational alterations to anxiety and depression-related behaviours of adult F1 and F2 offspring^15,16^.

It is unknown whether a shorter period of stress preconception could also influence offspring phenotypes. It is also uncertain whether intergenerational modifications of offspring behaviour manifest in mice, regardless of severity or chronicity of the paternal exposure. Thus, we investigated whether the subchronic CORT treatment of male C57Bl/6J breeders would impact offspring anxiety-related behaviours with the hypothesis that CORT treatment of male breeders for 7 days prior to conception would result in increased anxiety-like behaviours observed in their adult offspring.

## Results

### Physiological adaptations to subchronic CORT treatment

**Figure 1:**
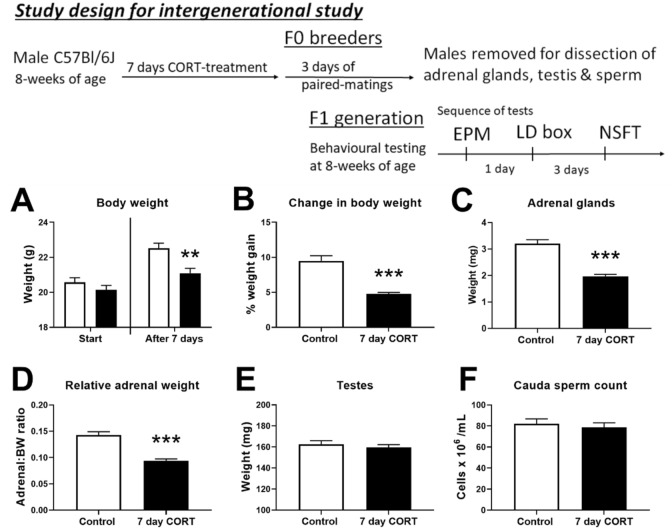
Physiological response of male breeders to CORT treatment. Schematic of study design displaying the sequences of experiments. 7 days of CORT supplementation impacted body weight gain of male breeders (**A**,**B**). Dissections performed after paired matings indicated diminishment of adrenal size (**C**,**D**). Testes size (**E**) and caudal epididymis sperm concentration (**F**) remained unaffected. The average overnight fluid intake for water controls was 4.03 ± 0.33 mL while the average intake for CORT-treated mice was 4.44 ± 0.19 mL (mean ± SEM; unpaired *t*-test: *p* = 0.313). n = 8 animals per group. Data presented as mean ± SEM and analysed by unpaired *t*-test **p < 0.01, ***p < 0.001.

All mice recorded week-on-week net weight gains, but CORT-treated mice weighed significantly less after 7 days (*p* < 0.01; Fig. 1A) due to reduced week-on-week percentage weight gain (*p* < 0.001; Fig. 1B). CORT treatment significantly reduced adrenal gland wet weight (*p* < 0.001; Fig. 1C), and that significant difference persisted after correcting for body weight (*p* < 0.001; Fig. 1D). There were no significant differences in testes wet weight (*p* = 0.531; Fig. 1E) nor in cauda epididymal sperm counts (*p* = 0.594; Fig. 1F). These results are consistent with the physiological adaptions in response to 28-day CORT treatment previously reported^16^.

### Subchronic CORT treatment does not affect anxiety-related behaviours

**Figure 2:**
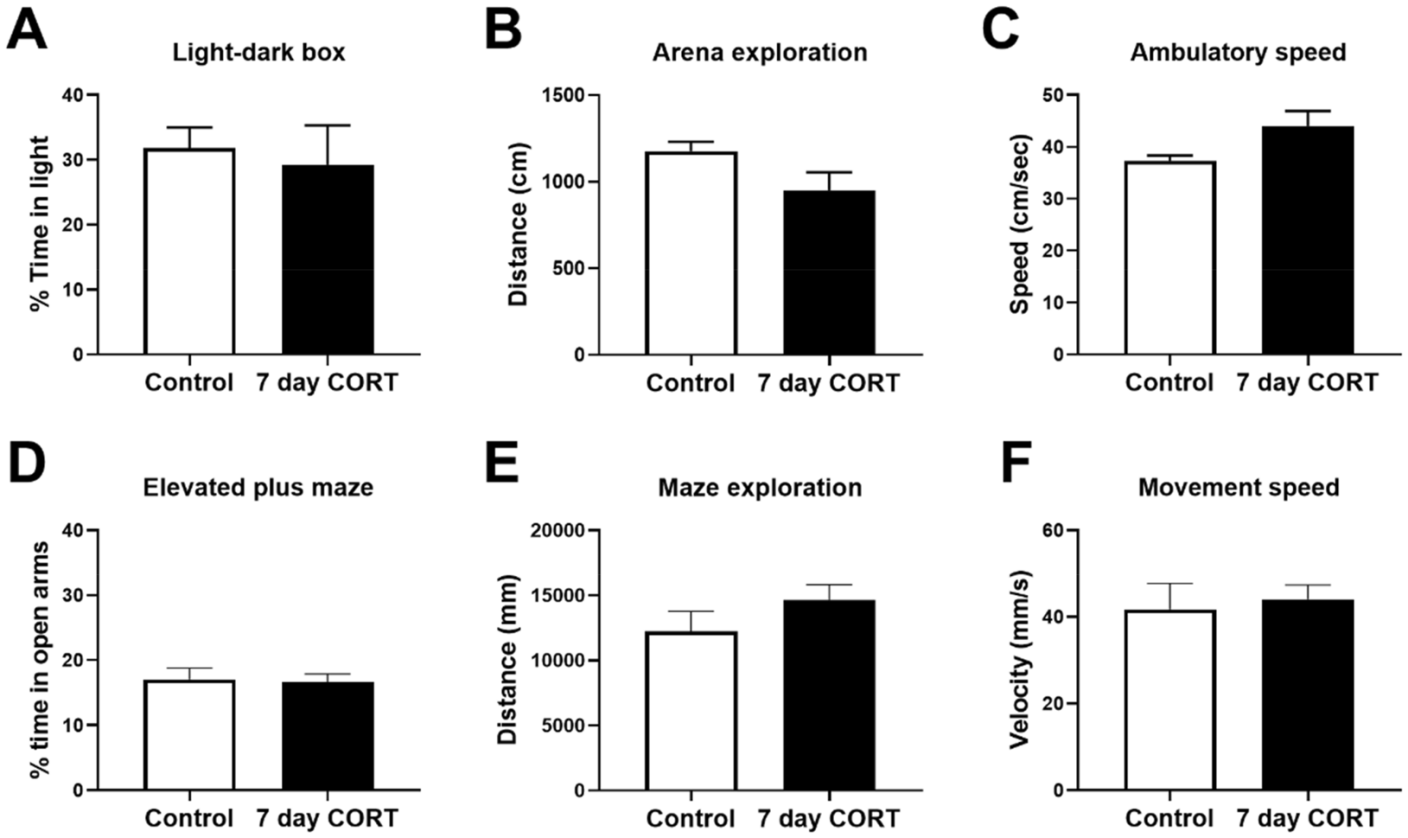
Anxiety-related behaviours are unaffected by 7 days of CORT treatment. CORT-treated mice did not significantly differ in the average time spent in the light (**A**), total distance moved (**B**) and the average ambulatory speed (**C**) in the light–dark box test. On the elevated-plus maze, no significant effect of CORT treatment was detected for time spent on open arms (**D**), total exploratory distance (**E**) and average movement speed (**F**). n = 5 animals per group. Data presented as mean ± SEM and analysed by unpaired *t*-test.

A separate cohort of mice that were not used for matings were assessed for potential behavioural effects of subchronic CORT treatment. There was no effect of CORT treatment on anxiety-related behaviour in the light–dark box (Fig. 2A–C) in the analysis of percentage time spent in the light compartment (t_(8)_ = 0.388, *p* = 0.708), exploratory distance (t_(8)_ = 1.921, *p* = 0.091), and average ambulatory speed (t_(8)_ = 2.159, *p* = 0.063). Similarly, on the elevated-plus maze (Fig. 2D–F), there was no significant differences in time spent on the open arms (t_(8)_ = 0.176, *p* = 0.864), exploratory distance (t_(8)_ = 1.25, *p* = 0.247), and average movement speed (t_(8)_ = 0.348, *p* = 0.737). The absence of any observable impacts on behaviour following CORT treatment is consistent with our previous study^16^.

### Subchronic CORT treatment affects hypothalamus gene expression

**Figure 3:**
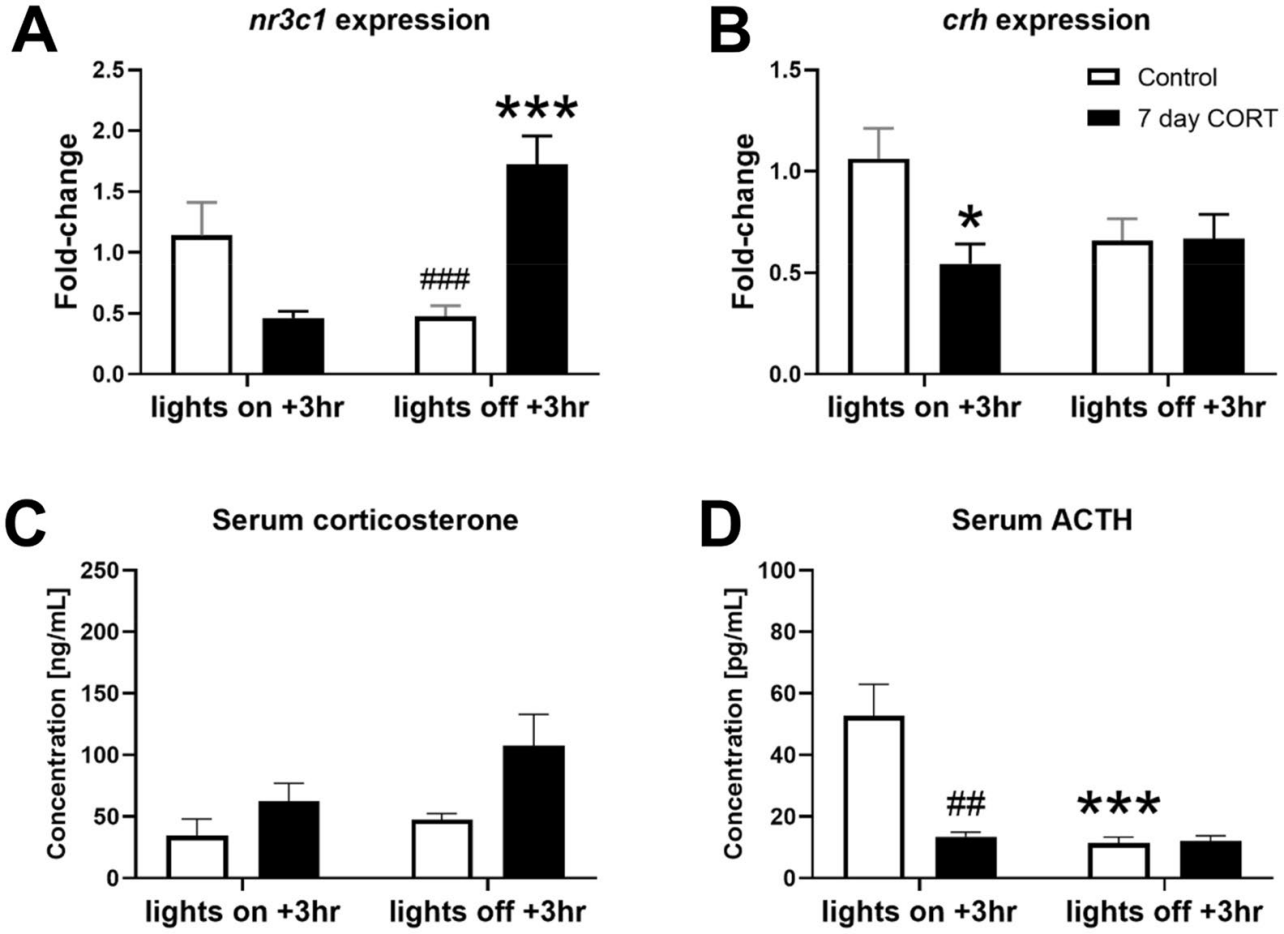
HPA axis activity is affected by subchronic CORT treatment. Gene expression profiling of the hypothalamus indicated dysregulation of the glucocorticoid receptor (**A**) and corticotrophin-releasing hormone (**B**). Serum CORT concentrations were increased overall (**C**) but ACTH concentrations were suppressed (**D**). Data expressed as mean ± SEM and analysed by two-way ANOVA. *denotes within time point comparisons and # denotes between time point comparisons with post-hoc *t*-tests: *p < 0.05, **p < 0.01, ***p < 0.001.

The diurnal rhythm of the HPA axis is well-established, partly involving interactions between CLOCK transcription factors and the glucocorticoid receptor^17,18^. Thus, we examined hypothalamus gene expression of glucocorticoid receptor (*NR3C1*) and corticotrophin-releasing hormone (*CRH*) at two distinct points of the light/dark cycle (‘lights on + 3 h’ and ‘lights off + 3 h’). There were no main effects of time (F_(1,26)_ = 2.591, *p* = 0.1196) or treatment group (F_(1,26)_ = 2.339, *p* = 0.1383) on *NR3C1* gene expression (Fig. 3A), but there was a significant time × group interaction (F_(1,26)_ = 26.88, *p* < 0.001). Post-hoc testing showed that *NR3C1* gene expression was significantly greater during the dark phase for CORT-treated mice only (*p* < 0.001). For *CRH* gene expression (Fig. 3B), there was no significant effect of time F_(1,26)_ = 1.378, *p* = 0.2511) but there was a significant effect of treatment (F_(1,26)_ = 4.514, *p* = 0.0433) and a significant time × treatment interaction (F_(1,26)_ = 4.835, *p* = 0.0370). Post-hoc testing showed that hypothalamic *CRH* expression was significantly lower in CORT-treated mice (*p* < 0.05) during the dark phase.

There was a significant treatment effect for serum CORT concentrations (F_(1,9)_ = 5.567, *p* = 0.0426) (Fig. 3C) but no significant effect of time (F_(1,9)_ = 2.433, *p* = 0.153). There was no significant time × treatment interaction (F_(1,9)_ = 0.7338, *p* = 0.4139) (Fig. 3C). Regarding serum ACTH concentrations, there were significant overall effects of treatment (F_(1,8)_ = 12.26, *p* = 0.0081) and time (F_(1,8)_ = 15.02, *p* = 0.0047) (Fig. 3D). There was also a significant time × treatment interaction (F_(1,8)_ = 13.03, *p* = 0.0069). Post-hoc comparisons indicated that serum ACTH concentrations of the control group at ‘lights on + 3 h’ were significantly higher compared to the CORT treated group at the same time (*p* < 0.01) and to the control group at ‘lights off + 3 h’ (*p* < 0.01).

### Subchronic CORT treatment does not affect epididymal GR expression in vivo

**Figure 4:**
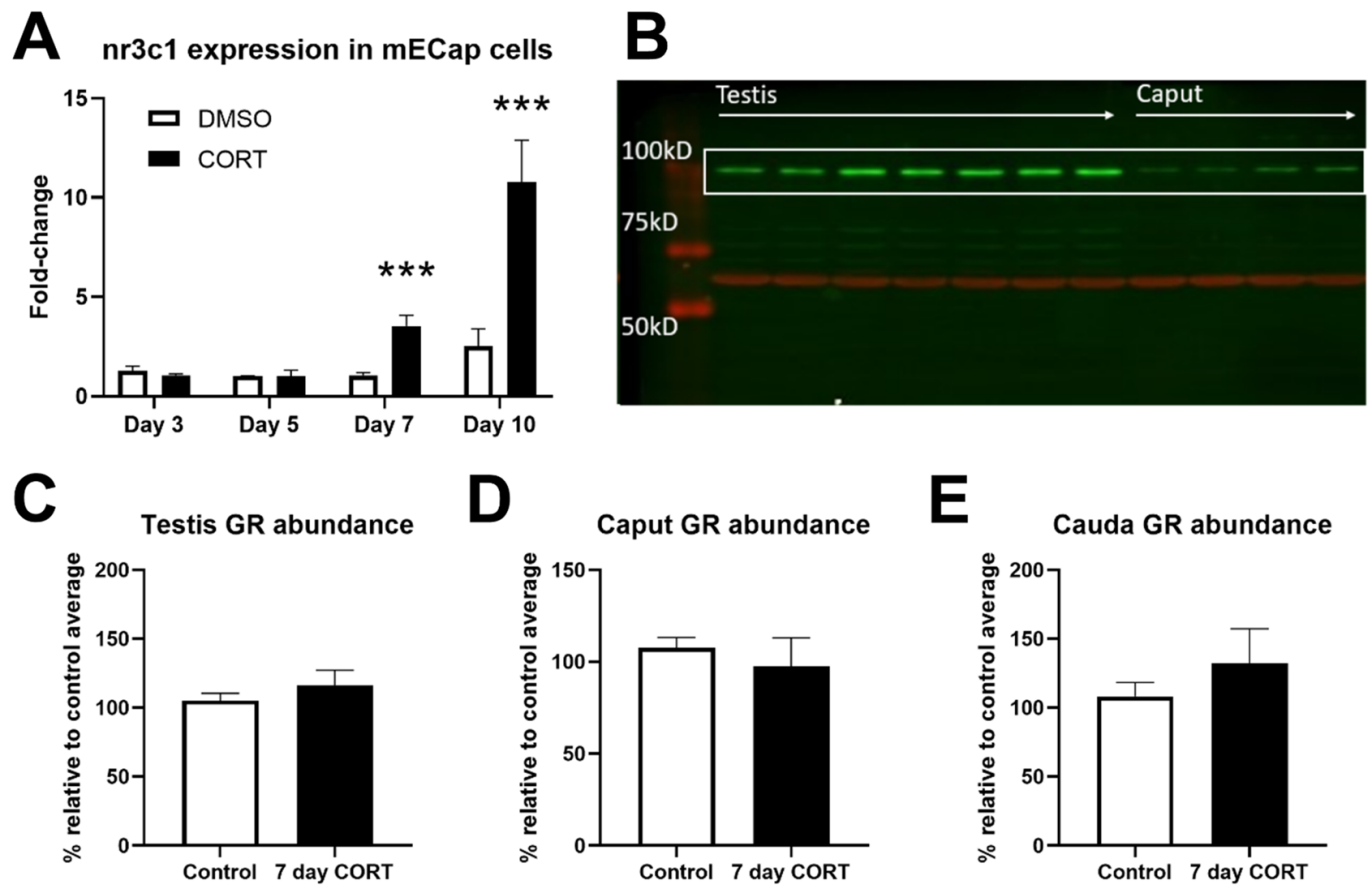
In vitro and in vivo responses of epididymal cells to CORT-treatment. mECap18 cells up-regulate *NR3C1* gene expression after 7 days of CORT treatment (**A**). Pooled data from 3 independent experiments comprising of 3 technical replicates per treatment for each time point. Gene expression data presented as mean fold-change ± SEM, analysed by unpaired *t*-test with corrections for multiple comparisons and adjusted α = 0.0125. A representative Western blot (**B**) showing GR-α at ~ 100kD and β-actin between 50 and 75 kD. Control and CORT samples were loaded sequentially. GR-α protein levels were unchanged in the testis (**C**), caput (**D**) and cauda epididymis (**E**) of animals after 7 days of CORT treatment. n = 5–6 animals per group. Data presented as mean ± SEM and analysed by unpaired *t*-test.

We previously proposed that the intergenerational effects of paternal stress involves molecular adaptation of epididymal endothelial cells to stress^19^. To test that hypothesis, we performed *NR3C1* gene expression profiling of an immortalized mouse caput epididymal epithelial cell line (mECap18) following direct stimulation with corticosterone. *NR3C1* gene expression did not differ between CORT-treated and DMSO-treated cells at the 3 (t_(16)_ = 1.06, *p* = 0.305) and 5 day (t_(16)_ = 0.0256, *p* = 0.980) timepoints but was significantly upregulated in CORT-treated cells at the 7 (t_(16)_ = 4.34, *p* = 0.001) and 10 day timepoints (t_(16)_ = 3.61, *p* = 0.002; Fig. 4A).

To determine the state of GR expression in vivo, we examined protein levels of GR-α (the dominant isoform in mice^20^ and humans^21^) in the male reproductive organs after 7-days of CORT treatment. No significant differences in GR-α protein levels (Fig. 4B) were detected between the groups for the testes (t_(9)_ = 0.838, *p* = 0.424; Fig. 4C), caput epididymis (t_(9)_ = 0.561, *p* = 0.588; Fig. 4D) and the cauda epididymis (t_(9)_ = 0.813, *p* = 0.437; Fig. 4E).

### Limited effects of subchronic paternal CORT treatment on offspring anxiety-relevant behaviours

Seven of the eight breeding dams in each treatment group (Control, PatCORT7) became pregnant and successfully littered down. One control dam ate her pups over the first days (leaving 2 alive) so this litter was excluded from the study. The remaining 6 litters yielded a total of 40 control F1 offspring (19 males, 21 females). A total of 55 PatCORT7 F1 offspring (27 males, 28 females) were obtained from the 7 PatCORT7 litters. Analysis of post-weaning growth indicated no differences in body weight gain up till 8 weeks of age (data not shown) when behavioural testing commenced.

**Figure 5:**
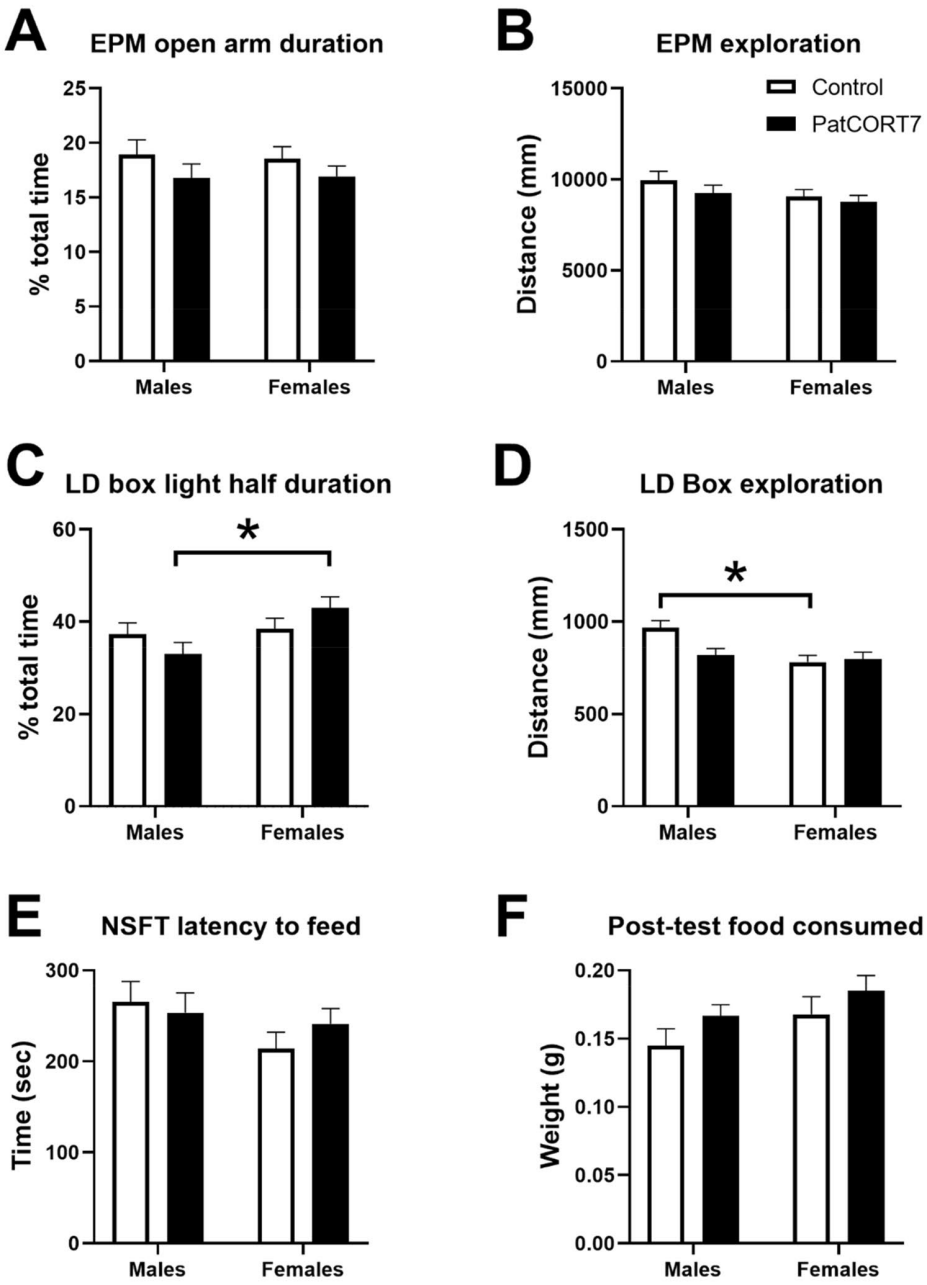
Intergenerational effects of paternal subchronic CORT treatment on F1 offspring anxiety. Comparing the behavioural responses of F1 offspring from control pairings and those sired by CORT-treated males, there were no differences on the elevated-plus maze (**A**,**B**), in the light–dark box (**C**,**D**) or in the novelty-suppressed feeding test (**E**,**F**). Data analysed by linear mixed models followed by post hoc tests where appropriate. *indicates post hoc testing *p* < 0.05. n = 19–28 mice per group. Data presented as mean ± SEM.

On the elevated-plus maze, there were no overall effects of sex (F_(1,86.688)_ = 0.0027, *p* = 0.959) or paternal treatment (F_(1,10.690)_ = 1.6885, *p* = 0.221) on the percentage of time spent by mice out on the open arms (Fig. 5A). The sex × paternal treatment interaction was not significant (F_(1,86.688)_ = 0.0313, *p* = 0.860). Regarding total distance moved in the elevated-plus maze (Fig. 5B), there were no effects of sex (F_(1,87.117)_ = 2.6477, *p* = 0.107) or paternal treatment (F_(1,11.275)_ = 0.7538, *p* = 0.403). There was no significant sex × paternal treatment interaction (F_(1,87.117)_ = 0.0755, *p* = 0.784).

In the light–dark box, for time spent in the light half, there was no effect of paternal treatment (F_(1,7.702)_ = 0.0380, *p* = 0.851) (Fig. 5C) but there was a significant sex difference (F_(1,86.232)_ = 4.5407, *p* = 0.0359). There was also a significant sex × paternal treatment interaction (F_(1,86.232)_ = 4.0764, *p* = 0.0466), with PatCORT7 females spending more time in the light than PatCORT7 males (*p* < 0.05). There was a significant effect of sex (F_(1,83.865)_ = 12.0017, *p* < 0.001) on total distance moved (Fig. 5D) but there was no effect on paternal treatment (F_(1,11.122)_ = 0.4289, *p* = 0.525). There was a significant sex × paternal treatment interaction (F_(1,83.865)_ = 8.3397, *p* = 0.00490), with control females moving lesser distances than control males (*p* < 0.001).

During the novelty-suppressed feeding test, there were no significant effects of sex (F_(1,86.815)_ = 2.3599, *p* = 0.128) or paternal treatment (F_(1,9.527)_ = 0.0282, *p* = 0.870) for latency to commence feeding (Fig. 5E). There was also no sex × paternal treatment interaction (F_(1,86.815)_ = 1.0582, *p* = 0.307). During the post-test feeding period (Fig. 5F), there were no differences in the amount of chow consumed associated with sex (F_(1,91)_ = 3.3953, *p* = 0.0686) or paternal treatment (F_(1,91)_ = 3.0409, *p* = 0.0846), and there was no interaction (F_(1,91)_ = 0.0345, *p* = 0.8530).

## Discussion

Our results indicated that subchronic treatment of adult male C57Bl/6J mice with low-dose CORT caused minimal physiological changes with no impact on anxiety-related behaviours. Their fertility was also unaffected, which allowed the generation of their offspring with no difficulty. Subsequently, we found that the short period of treatment prior to mating was insufficient to induce an intergenerational shift in offspring anxiety-related behaviours. We have provided initial in vitro evidence that mouse epididymal cells are responsive to CORT but that response was not replicated in vivo. Given our previous report that chronic (28 day) paternal CORT treatment is associated with behavioural modifications in offspring^15,16^, this study has now demonstrated that the duration of stress exposure is a crucial determinant of the paternal-derived intergenerational risk for anxiety disorders.

This prompts further questions regarding the discrete influences of periodicity, age of exposure, and stress severity on the observable intergenerational effects. There is still a lack of comprehensive understanding in this regard as there have been relatively few intergenerational mammalian studies of paternal stress published (and primarily in mice). Closer comparisons of those studies could identify subtle differences in the intergenerational outcomes that are likely attributable to distinct differences in experimental protocols, and standardisation should certainly be a priority within the field. For example, unlike the absence of behavioural deficits caused by subchronic CORT treatment, 10 days of chronic social defeat (CSD) leads to the development of robust behavioural deficits in adult mice such as social withdrawal, hypolocomotion, pro-anxiety and depressive-like behaviours^22–24^. CSD also gives rise to neuroendocrine dysregulation including suppression of HPA axis activity^25–28^, which has not been observed to be impacted by CORT treatment^29^. In contrast to this study, paternal CSD is associated with pro-anxiety and depressive-like behavioural modifications in male and female F1 offspring^8^. Given the stark differences in the behavioural and neuroendocrine effects on the male breeders despite the relatively similar durations of exposure, it is therefore reasonable to propose that the severity of paternal stress experienced is a crucial factor in determining the extent to which offspring mental health is intergenerationally affected. However, one limitation to our study was the small sample size used to establish the direct impact of subchronic CORT treatment on behaviour. This aspect could be investigated more thoroughly in future, especially since we have now identified differences in gene expression at two distinct time points of the day/light cycle, and thus the modulatory impacts of circadian rhythms could be explored in future studies.

One other possible reason for the disparity between our present findings and paternal CSD studies could be differences in breeding strategies. While our breeding protocols pair-mate CORT-treated males immediately after treatment, Dietz and colleagues permitted CSD breeders a 1-month recovery period prior to mating^8^. We performed sperm counts to verify that fertility was unlikely to be affected in our model, and our breeding outcomes suggest no negative impact on fecundity. It is unknown if fecundity would be detrimentally affected in the immediate period after CSD stress (i.e. in the 5 days after cessation of CSD), or if the reported intergenerational effects of CSD would also manifest in the offspring conceived during that immediate period post-stress. We cannot exclude the possibility that our subchronic CORT treatment could still harbour intergenerational effects that would only manifest after a further incubation period. Future studies could incorporate a post-treatment recovery period aligned to the mouse spermatogenic cycle (~ 42 days) before mating for the F1 generation. A very recent study reported that effects of 28 days of paternal chronic stress had intergenerational effects after 12 weeks of post-stress recovery but not after 1 week^30^. That extended period exceeds several spermatogenic cycles, and when allowing for the elimination of mature epididymal sperm, it suggests that a life-long imprint of that history of paternal stress is likely to originate from testicular germ cells through more stable epigenetic modifications, such as DNA methylation or other alterations in various somatic cells that maintain differences in non-coding RNAs in germ cells. It would be interesting to investigate if and how varying lengths of CORT-treatment affect testis DNA methylation, and if F1 offspring behavioural phenotypes differentially manifest with post-treatment recovery periods. A further consideration would be the extent to which sperm are exposed to CORT in our model, which is dependent on the epididymal transit time of mouse sperm—estimated to be up to 10 days (interestingly only 2–6 days for humans). Since we have shown that mECap18 cells only begin to adapt to CORT exposure after 7 days, it is likely that the F1 offspring we studied are produced by sperm affected by this subchronic CORT treatment (i.e. prior to epididymal epithelial cells undergoing allostatic adaptation). The decision of when matings occur is one aspect of experimental design worth deeper consideration because of the translational value of our animal models. Moderate stress and negative emotions in men have been associated with a paradoxical increase in sexual interest^31^. However, the presence of more severe mood disorders is linked to a decrease in sexual activity^32^. There is some evidence that moderate stress-associated increases in sexual activity also exist for young adult women, but the participant age range in this one study was highly restrictive (18–20 years)^33^. Thus, if couples are more likely to conceive whilst one or both of them are under stress, then it would be important to factor this into future experimental designs and clarify the potential intergenerational implications for the health of those children.

In mice, the age at which the male is exposed to stressors is predictive of behavioural deficits developing in his offspring. For example, modelling early life adversity in mice through daily post-natal maternal separation (of at least 3 h) leads to pro-anxiety and depressive-like behaviours^34,35^ but does not affect the same spectrum of behaviours in F1 offspring^36^. However, Rodgers et al.^9^ reported that the intergenerational blunting effect on F1 offspring stress response was similar regardless of whether breeders were exposed to chronic variable stress during adulthood only (8–14 weeks of age) or if the stress commenced from 4 weeks of age (prepuberty). Interestingly, chronic social instability stress starting from a juvenile age till adulthood also induces transgenerational modifications to offspring behaviour and, in contrast to the majority of paternal stress models studied to-date, only female F1 offspring displayed pro-anxiety behaviours and social deficits with the males relatively spared^37^. It was noted that only the male F1 germ line transmitted these effects to the F2 and F3 generations, and this strengthens the notion that transgenerational effects are indeed transmitted via the male germ line. Social instability stress also changed the expression profile of sperm miRNAs^38^, which interestingly included miRNAs (e.g. MIR-449a) previously identified as being affected by chronic CORT treatment^16^. One reason for preadolescent stress having transgenerational effects in that series of studies could have been the nature of the stressor. Juvenile and adolescent mice naturally display high levels of sociability and reach a peak of social development by 49-days of age^39^. Thus, repeated disruption of this critical period by changing cage mates twice weekly could have direct consequences for the normal development of the HPA and hypothalamus–pituitary–gonadal (HPG) axes. It would be interesting if aspects of sexual maturation and male fertility would be further verified in this model. More recently, one study reported that a slight modification to the chronic social instability protocol (every 3 days for 7 weeks) was effective for inducing pro-anxiety and depressive-like behaviours in adult male mice^40^, so intergenerational modifications could be further investigated in that model.

While it is unknown whether different intergenerational outcomes are dependent on the age at which CORT treatment commences (juvenile vs adult), such a comparison would be challenging due to the established differences in the physical adaptative responses to chronic low-dose CORT treatment by adolescent (4 weeks of age) and adult (10 weeks of age) mice^29^. Any intergenerational effects observed would be confounded by secondary effects on the physical and sexual maturation of adolescent animals. It would, however, be interesting to investigate whether a limited 1-week CORT treatment of adolescent animals post-weaning could have intergenerational effects.

To our knowledge, this is the first study of how GR expression in male reproductive organs is regulated by stress. While no significant differences were observed in vivo, it remains to be determined whether chronic CORT treatment could alter GR expression in the testes and epididymides, or perhaps shift the sensitivity of those organs to glucocorticoids. Previous research had implicated differential expression of GR protein in the hippocampus as a key distinction between states of susceptibility and resilience. The susceptibility of an individual mouse to 10 days of social defeat stress can be predicted by the extent to which GR protein levels are moderated in the hippocampus^41^. That time-dependent shift in GR expression is key to allostasis whereby the repeated or accumulated exposure to stress renders an individual more susceptible to mental illness (allostatic load)^42^. A shift in the allostatic threshold is possibly observed in the delayed response of mECap18 cells in vitro, suggestive of a gradual shift in sensitivity to CORT. While we have observed strikingly different in vitro and in vivo effects, our in vitro result is consistent with an independent study that applied CORT of a similar concentration to a different caput cell line (DC-2)^30^. One possible reason for the non-effect in vivo is the presence of physical barriers (such as the blood-testis barrier) that protect the epididymal cells from direct unregulated interactions with CORT. Of course there are other major differences, the most prominent being that the epididymal microenvironment and physiological blood perfusion cannot be mimicked under in vitro conditions. There are many autonomic and sympathetic adaptations in a stressed animal that could influence reproductive function, including the up-regulation of secondary regulatory molecules that are distributed in plasma-borne microvesicles^43–45^. Further studies will be required to elucidate these factors.

It is surprising how little is known of the physiological and molecular adaptation of the male reproductive organs to stress. While epididymal expression of other steroid receptors such as the estrogen receptor have been reported on^46^, the expression profile of the GR isoforms across the epididymal segments is unclear. Accordingly, their regulation under conditions of psychological or physical stress are also unknown. In this study, we quantified protein levels of GRα, the key isoform of GR that is crucially involved in transactivation, subcellular localization and the initiation of key molecular signalling pathways^47–50^. We analysed Western blot data from whole tissue lysates aiming at capturing information regarding the other GR isoforms that exist (some still of unknown function). However, due to inconsistencies in the detection of the minor bands and generally low abundance of those isoforms, we excluded this data from further analysis. Factoring in the low abundance of protein will be essential in the design of future studies. A further refinement would be to attempt a quantification study on the separate subcellular fractions. One motivation for that would be a previous report that the cytoplasmic and nuclear protein fractions extracted from the placentae of male and female foetuses contain at least eight known GR isoforms in the mouse placenta^20^. This finding was consistent with human and guinea pig placenta^51^ suggesting conservation of GR expression patterns across species. Therefore, further characterisation of the GR expression in the male reproductive organs of rodents could be highly informative for human biology.

Our collective work emphasizes that not all paternal exposures to stress translate to negative health outcomes for offspring. We have demonstrated major differences in the intergenerational consequences of subchronic and chronic stress exposures, in addition to age of exposure and severity of stress. There is poor understanding of the physiological and molecular adaptations of the male reproductive organs to stress. Ultimately, it is vital that we unravel the complexity of the sperm epigenome and its regulation by stress since sperm are effectively the final messengers of paternal health status, delivering their genetic and epigenetic information at conception to the next generation.

## Methods

### Animals and housing conditions

Seven-week-old male and female C57Bl/6J mice were purchased from the Animal Resource Centre (Perth, WA). Mice were acclimatised to the Florey Institute animal facility for 1 week prior to commencement of the study. Subsequently, animals were randomly allocated to either the subchronic stress group or control group. Male mice assigned to the subchronic stress group were provided drinking water supplemented with corticosterone hemisuccinate (25 mg/L; Steraloids Inc, RI, USA) that was prepared twice weekly, as previously published^16^. Mice comprising the control group received untreated filtered tap water. Food and fluids were provided ad libitum. Mice were housed in the Florey Institute animal facility in a room maintained at 22–25 °C/55% humidity on a 12:12H light–dark cycle (lights on at 0700H). Male and female breeders were single housed in cages with wood chip bedding and provided tissue paper as nesting material prior to paired matings. After 7 days of treatment, mice were pair-mated for 3 days to generate the F1 offspring during which time untreated water was provided. Treated mice that were not pair-mated were used for behavioural testing, serum assays and gene expression profiling studies. After the pairing period, females were separated into single-housing and left undisturbed apart from weekly box changes until they littered down. F1 offspring were weaned at 28 days of age and sex-sorted into groups of 3–5 mice per cage within each paternal treatment group. They were left to age undisturbed until 8 weeks of age when behavioural testing commenced.

### Behavioural tests

Tests were performed in a room different from the housing room. All mice were allowed to acclimatize to the testing room for at least 30 min prior to commencement of each test. Testing was conducted between 0800 and 1300 h and under dimmed lighting of 30–50 lx. Animals were tested in a randomised order with the experimenter blind to the paternal condition. The sequence of tests was the same for all subjects.

Elevated-plus maze was performed as previously described^16^. Briefly, maze arms were 30 cm in length and 5 cm in width, and the closed arms had walls of 14 cm height. The activity pattern of each mouse over 5 min was automatically tracked from overhead using the CleverSys TopScan software (Reston, VA, USA). Total time spent in the open arms was expressed as a percentage of the test duration.

Light–dark box was performed as previously described^16^. Briefly, the test was conducted using the Med Associates ENV-510/511 open-field arenas (Fairfax, VT, USA). The light compartment was illuminated with overhead LED lamps to 750 lx. Mice started each 10-min test session in the dark insert. Time spent in each half of the arena was automatically tabulated by the proprietary activity monitor software and expressed as a percentage of the test duration.

Novelty-suppressed feeding test (NSFT) was performed as previously described^15^. Briefly, mice were food-deprived for 48 h with a 1 h feeding period after the first 24 h. Mice were weighed on the morning of the NSFT to calculate % body weight loss, with an expected weight loss of at least 15%. All mice met this criterion. Testing was conducted under dimmed lighting of 25 lx in a 30 × 30 × 30 cm novel arena lined with clean bedding with a food pellet in the centre. The latency for the mouse to assume a stationary posture while feeding on the pellet was recorded. When this occurred, the mouse was immediately removed and placed into a clean cage containing a pre-weighed food pellet for 10 min. The amount consumed during that period was recorded. All mice were then returned to their original boxing configurations with *ab libitum* food and water access.

### Tissue and serum collection

Mice were killed by cervical dislocation and trunk blood was collected. For the gene expression studies, mice were killed for brain dissections at 2 distinct points of the day/light cycle (+ 3 h lights on ~ 10am and + 3 h lights off ~ 10 pm). Brains were immediately removed from the skull and the hypothalamus was fresh dissected on ice, snap frozen in liquid nitrogen then transferred to storage at − 80 °C until further use. Whole blood was left to clot for 30 min before separation of serum by centrifugation at 1100 g for 15 min, which was stored at − 20 °C until subsequent analysis. Corticosterone concentrations were assayed using an EIA as per manufacturer’s instructions (#501320, Caymen Chemical, Ann Arbor, MI, USA). ACTH concentrations were determined by a commercial service provider (Cardinal Bio-Research, New Farm, QLD, Australia) using a Millipore Luminex platform with the MILIPLEX MAP mouse bone panel (MBNMAG-41K). One control sample from the ‘lights on + 3 h’ group failed and was excluded from the analysis.

### Collection of sperm

Cauda epididymides were separated from the corpus segment and the epididymal fat pad was removed, leaving only the vas deferens attached. The cauda epididymides were bisected with a single incision then immersed into 1 mL of M2 medium (M7167, Sigma-Aldrich, NSW, Australia) pre-warmed to 37 °C. After at least 30 min incubation at 37 °C, tissue was discarded and spermatozoa were resuspended. An aliquot was removed and stained with Trypan Blue for the purpose of cell counting. Sperm were pelleted and archived at − 80 °C for separate studies.

### mECap18 cell culture and corticosterone treatment

The SV40-immortalized mouse caput epididymal epithelial (mECap18) cell line was provided as a kind gift from Dr. Petra Sipila (Turku University, Turku, Finland). mECap18 cells were cultured in DMEM (4.5 g/L glucose) supplemented with 100 μM sodium pyruvate, 200 μM L-glutamate, 100 U/mL penicillin, 10 μg/mL streptomycin, and 10% v/v heat inactivated fetal bovine serum with 50 nM 5α-androstan-17β-ol3-one with passaging as required^52^. Cells were plated and allowed to grow for 72 h prior to treatment with 100 nM 4-pregnen-11β, 21-diol-3, 20-dione 21-acetate (Q1570-000, Steraloids Inc, Rhode Island, USA) for up to 10 days with media replacement occurring at intervals of 48 h. Following treatment, cells were harvested at days 3, 5, 7 and 10 prior to performing RNA extraction. Briefly, total RNA was isolated by homogenisation of mECap18 cells in Solution D (4M guanidium thiocyanate, 25 mM sodium citrate, 0.5% sarkosyl, 0.72% 2-mercaptoethanol). RNA was subsequently extracted using a standard phenol/chloroform protocol before being precipitated with isopropanol. The RNA pellet was then redissolved in Solution D and precipitated with ethanol. Finally, the pellet was air dried and dissolved in RNase-free water.

### Western blot for glucocorticoid receptor protein levels

Whole epididymides were dissected from an independent group of CORT treated and control mice, and trisected to obtain the caput, corpus and cauda segments of the epididymides. These were frozen on dry ice then stored at − 80 °C. Frozen tissue was homogenised in a cold RIPA buffer (10 mM tris pH 7.5, 80 mM NaCl, 0.5% IGEL-PAL, 0.05% SDS, 0.3% sodium deoxycholate and protease inhibitor cocktail (Sigma, Cat# P8340)) with sonication. Samples were centrifuged at 10,000 g for 10 min at 4 °C. The supernatant concentration was determined using the Pierce Bicinchoninic Acid (BCA) Protein Assay Kit (Cat# 23227, Thermo Fisher Scientific). 30 µg of protein was then loaded into each lane of a 10% SDS PAGE gel. After protein separation, the proteins were transferred on to a PVDF membrane and blocked with Odyssey Blocking Buffer solution (LI-COR Biotechnology, Cat# 927-40000) for 30 min. Membranes were incubated overnight at 4 °C with primary antibodies (Cat# HPA004248, Rabbit anti-NR3C1 antibody, Sigma-Aldrich; Cat# 4967, mouse β-Actin antibody, Cell Signalling) in the blocking buffer. Secondary antibodies used for detecting signals were IRDye 800CW Goat anti-Rabbit IgG Secondary Antibody and IRDye 680LT Goat anti-Mouse IgG Secondary Antibody (Licor). Total relative protein abundances were determined using the Pierce Reversible Protein Stain Kit (Cat# 24580, Thermo Fisher Scientific). Blots were analysed by Image Studio Lite (Version 5.2.5) and ImageJ ver 1.52q. Data was expressed as a percentage of the maximum signal of the target protein on each membrane.

### Gene expression studies

Frozen hypothalamus tissue was homogenised in Qiazol lysis buffer using a Diagenode bioruptor (UCD-300, Liege, Belgium), then total RNA was isolated using RNeasy Mini Kits as per the manufacturer’s instructions (QIAGEN, Chadstone, VIC, Australia), including on-column DNaseI digestion. Concentration and quality of the eluted RNA was determined using Nanodrop 2000c spectrophotometer (Thermo Scientific, Wilmington, DE, USA) and Agilent 2100 Bioanalyser Nanochips (Cat# 5067; Agilent Technologies; Santa Clara, CA, USA). 1 μg of total RNA was reverse transcribed into cDNA using Invitrogen Superscript VILO cDNA synthesis kit (Cat#11754050; Life Technologies Australia). The cDNA was then diluted 50 × prior to qPCR. Primer sequences were as previously published^53^. qPCR reactions were performed in an Agilent Biosystems ViiA7 platform using technical triplicates.

For the in vitro study on mECap18 cells, total RNA was treated with RQ1 RNase-free DNase (M6101; Promega, Madison, WI, USA) to remove genomic contamination. 2 μg of total RNA was then reverse transcribed using 500 ng oligo dT, 5 × buffer, 100 mM DTT, 40 U of rRNasin (N2111; Promega, NSW, Australia), 0.5 mM dNTPs, and 20 U of M-MLV reverse transcriptase (M1701; Promega, NSW, Australia). Primer sequences were as follows: NR3C1, forward (5′ GAGGACAACCTGACTTCCTTGGG 3′), reverse (5′ GTGGTCCCGTTGCTGTGGA 3′) and cyclophilin, forward (5′ CGTCTCCTTCGAGCTGTTT 3′), reverse (5′ ACCCTGGCACATGAATCCT 3′). Real-time analysis was performed on a Roche Light Cycler 96 SW 1.1 using Promega GoTaq qPCR mastermix (M7122; Promega, NSW, Australia) according to the manufacturer’s instructions. Each data set represents 3 independent replicates of treated cells. All PCR products were sequenced to ensure correct sequence amplification. Cyclophilin was used as the endogenous reference gene. Relative gene expression was calculated using the -∆∆C_T_ method.

### Statistical analysis

Data was analysed in Graphpad Prism (v8.3.0 for Windows, GraphPad Software, San Diego, CA, USA) or Ryy^54^. Unpaired *t*-tests were used to analyse the physiological parameters and behavioural data for male breeders. 2-way ANOVA was used to analyse the hypothalamus and serum data sets with ‘treatment’ and ‘time’ as the fixed between-subject factors. Sidak post hoc testing, where appropriate, incorporated corrections for multiple comparisons. Offspring behavioural data was analysed by linear mixed models with ‘sex’ and ‘paternal treatment’ as fixed between-subject factors effects and ‘litter’ as random effect. If there were significant main or interaction effects, post hoc pairwise comparisons were computed from the contrasts between factors using the lsmeans package with Tukey adjustments. To meet the assumptions of parametric analysis, the Shapiro–Wilk test was applied. All main effects and interaction terms were tested on local significance level α = 0.05. Graphs were created using GraphPad Prism.

### Ethics statement

All animal experiments were approved by the Florey Institute Animal Ethics Committee and conducted in accordance with National Health and Medical Research Council of Australia guidelines and the Australian code for the care and use of animals for scientific purposes.

## References

[1] Bowers ME, Yehuda R (2016). Intergenerational transmission of stress in humans. Neuropsychopharmacology.

[2] Yehuda R (2000). Low cortisol and risk for PTSD in adult offspring of holocaust survivors. Am. J. Psychiatry.

[3] Yehuda R, Halligan SL, Bierer LM (2002). Cortisol levels in adult offspring of Holocaust survivors: relation to PTSD symptom severity in the parent and child. Psychoneuroendocrinology.

[4] Yehuda R (2007). Parental posttraumatic stress disorder as a vulnerability factor for low cortisol trait in offspring of holocaust survivors. Arch. Gen. Psychiatry.

[5] Duarte CS (2006). Posttraumatic stress in children with first responders in their families. J. Trauma Stress.

[6] Uchida M (2018). Parental posttraumatic stress and child behavioral problems in world trade center responders. Am. J. Ind. Med..

[7] Brand SR, Engel SM, Canfield RL, Yehuda R (2006). The effect of maternal PTSD following in utero trauma exposure on behavior and temperament in the 9-month-old infant. Ann. N. Y. Acad. Sci..

[8] Dietz DM (2011). Paternal transmission of stress-induced pathologies. Biol. Psychiatry.

[9] Rodgers AB, Morgan CP, Bronson SL, Revello S, Bale TL (2013). Paternal stress exposure alters sperm microRNA content and reprograms offspring HPA stress axis regulation. J. Neurosci..

[10] Rodgers AB, Morgan CP, Leu NA, Bale TL (2015). Transgenerational epigenetic programming via sperm microRNA recapitulates effects of paternal stress. Proc. Natl. Acad. Sci. USA.

[11] Ketchesin KD, Stinnett GS, Seasholtz AF (2017). Corticotropin-releasing hormone-binding protein and stress: from invertebrates to humans. Stress.

[12] Romero LM, Gormally BMG (2019). How truly conserved is the "well-conserved" vertebrate stress response?. Integr. Comp. Biol..

[13] Karatsoreos IN (2010). Endocrine and physiological changes in response to chronic corticosterone: a potential model of the metabolic syndrome in mouse. Endocrinology.

[14] Du X, Pang TY (2015). Is Dysregulation of the HPA-axis a core pathophysiology mediating co-morbid depression in neurodegenerative diseases?. Front. Psychiatry.

[15] Rawat A, Guo J, Renoir T, Pang TY, Hannan AJ (2018). Hypersensitivity to sertraline in the absence of hippocampal 5-HT1AR and 5-HTT gene expression changes following paternal corticosterone treatment. Environ. Epigenet..

[16] Short AK (2016). Elevated paternal glucocorticoid exposure alters the small noncoding RNA profile in sperm and modifies anxiety and depressive phenotypes in the offspring. Transl. Psychiatry.

[17] Nader N, Chrousos GP, Kino T (2010). Interactions of the circadian CLOCK system and the HPA axis. Trends Endocrinol. Metab..

[18] Nicolaides NC, Charmandari E, Chrousos GP, Kino T (2014). Circadian endocrine rhythms: the hypothalamic-pituitary-adrenal axis and its actions. Ann. N. Y. Acad. Sci..

[19] Pang TYC, Short AK, Bredy TW, Hannan AJ (2017). Transgenerational paternal transmission of acquired traits: Stress-induced modification of the sperm regulatory transcriptome and offspring phenotypes. Curr. Opin. Behav. Sci..

[20] Cuffe JSM, Saif Z, Perkins AV, Moritz KM, Clifton VL (2017). Dexamethasone and sex regulate placental glucocorticoid receptor isoforms in mice. J. Endocrinol..

[21] Pujols L (2002). Expression of glucocorticoid receptor alpha- and beta-isoforms in human cells and tissues. Am. J. Physiol. Cell Physiol..

[22] Harris AZ (2018). A novel method for chronic social defeat stress in female mice. Neuropsychopharmacology.

[23] Menard C (2017). Social stress induces neurovascular pathology promoting depression. Nat. Neurosci..

[24] Nakatake Y (2019). The effects of emotional stress are not identical to those of physical stress in mouse model of social defeat stress. Neurosci. Res..

[25] Bowens N, Heydendael W, Bhatnagar S, Jacobson L (2012). Lack of elevations in glucocorticoids correlates with dysphoria-like behavior after repeated social defeat. Physiol. Behav..

[26] Hartmann J (2012). Fkbp52 heterozygosity alters behavioral, endocrine and neurogenetic parameters under basal and chronic stress conditions in mice. Psychoneuroendocrinology.

[27] Hartmann J (2012). The involvement of FK506-binding protein 51 (FKBP5) in the behavioral and neuroendocrine effects of chronic social defeat stress. Neuropharmacology.

[28] Lehmann ML, Mustafa T, Eiden AM, Herkenham M, Eiden LE (2013). PACAP-deficient mice show attenuated corticosterone secretion and fail to develop depressive behavior during chronic social defeat stress. Psychoneuroendocrinology.

[29] Shahanoor Z, Sultana R, Baker MR, Romeo RD (2017). Neuroendocrine stress reactivity of male C57BL/6N mice following chronic oral corticosterone exposure during adulthood or adolescence. Psychoneuroendocrinology.

[30] Chan JC (2020). Reproductive tract extracellular vesicles are sufficient to transmit intergenerational stress and program neurodevelopment. Nat. Commun..

[31] Bancroft J (2003). The relation between mood and sexuality in heterosexual men. Arch. Sex. Behav..

[32] Gaskins AJ, Sundaram R, Buck Louis GM, Chavarro JE (2018). Predictors of sexual intercourse frequency among couples trying to conceive. J. Sex. Med..

[33] Hall KS, Kusunoki Y, Gatny H, Barber J (2014). Stress symptoms and frequency of sexual intercourse among young women. J. Sex. Med..

[34] Millstein RA, Holmes A (2007). Effects of repeated maternal separation on anxiety- and depression-related phenotypes in different mouse strains. Neurosci. Biobehav. Rev..

[35] Romeo RD (2003). Anxiety and fear behaviors in adult male and female C57BL/6 mice are modulated by maternal separation. Horm. Behav..

[36] Weiss IC, Franklin TB, Vizi S, Mansuy IM (2011). Inheritable effect of unpredictable maternal separation on behavioral responses in mice. Front. Behav. Neurosci..

[37] Saavedra-Rodriguez L, Feig LA (2013). Chronic social instability induces anxiety and defective social interactions across generations. Biol. Psychiatry.

[38] Dickson DA (2018). Reduced levels of miRNAs 449 and 34 in sperm of mice and men exposed to early life stress. Transl. Psychiatry.

[39] Nardou R (2019). Oxytocin-dependent reopening of a social reward learning critical period with MDMA. Nature.

[40] Yohn CN (2019). Social instability is an effective chronic stress paradigm for both male and female mice. Neuropharmacology.

[41] Han QQ (2017). Differential GR expression and translocation in the hippocampus mediates susceptibility vs. resilience to chronic social defeat stress. Front. Neurosci..

[42] McEwen BS (2005). Stressed or stressed out: what is the difference?. J. Psychiatry Neurosci..

[43] Fleshner M, Crane CR (2017). Exosomes, DAMPs and miRNA: features of stress physiology and immune homeostasis. Trends Immunol..

[44] Saeedi S, Israel S, Nagy C, Turecki G (2019). The emerging role of exosomes in mental disorders. Transl. Psychiatry.

[45] Vulpis E, Soriani A, Cerboni C, Santoni A, Zingoni A (2019). Cancer exosomes as conveyors of stress-induced molecules: new players in the modulation of NK cell response. Int. J. Mol. Sci..

[46] Hess RA (2011). Estrogen and its receptors in efferent ductules and epididymis. J. Androl..

[47] Nicolaides N, Lamprokostopoulou A, Sertedaki A, Charmandari E (2016). Recent advances in the molecular mechanisms causing primary generalized glucocorticoid resistance. Hormones (Athens).

[48] Patel R, Williams-Dautovich J, Cummins CL (2014). Minireview: new molecular mediators of glucocorticoid receptor activity in metabolic tissues. Mol. Endocrinol..

[49] Scheschowitsch K, Leite JA, Assreuy J (2017). New insights in glucocorticoid receptor signaling-more than just a ligand-binding receptor. Front. Endocrinol. (Lausanne).

[50] Wilkinson L, Verhoog N, Louw A (2018). Novel role for receptor dimerization in post-translational processing and turnover of the GRalpha. Sci. Rep..

[51] Saif Z (2015). Expression of eight glucocorticoid receptor isoforms in the human preterm placenta vary with fetal sex and birthweight. Placenta.

[52] Zhou W, Sipila P, De Iuliis GN, Dun MD, Nixon B (2018). Analysis of Epididymal protein synthesis and secretion. J. Vis. Exp..

[53] Du X (2012). Environmental enrichment rescues female-specific hyperactivity of the hypothalamic-pituitary-adrenal axis in a model of Huntington's disease. Transl. Psychiatry.

[54] R Development Core Team. *R: a language and environment for statistical computing*. https://www.R-project.org (2013).

